# Centenarians of the Basque Country are resilient to cancer

**DOI:** 10.1007/s11357-024-01425-4

**Published:** 2024-11-09

**Authors:** Sara Cruces-Salguero, Igor Larrañaga, Javier Mar, Ander Matheu

**Affiliations:** 1 Cellular Oncology Group, Biogipuzkoa Health Research Institute, Paseo Dr. Beguiristain S/N, 20014 San Sebastian, Spain; 2 Osakidetza Basque Health Service, Debagoiena Integrated Healthcare Organisation, Research Unit, Arrasate-Mondragón, Gipuzkoa, Spain; 3 Kronikgune Institute for Health Services Research, Barakaldo, Spain; 4 Biogipuzkoa Health Research Institute, San Sebastian, Gipuzkoa, Spain; 5 IKERBASQUE, Basque Foundation for Science, Bilbao, Spain; 6 Centro de Investigación Biomédica en Red de Fragilidad y Envejecimiento (CIBERfes), Carlos III Institute, Madrid, Spain

**Keywords:** Tumors, Resistance, Metastasis, Survival

## Abstract

Cancer is one of the leading causes of death and its prevalence increases with age. While centenarians exhibit extreme longevity and potential to avoid or delay aging-related diseases, their response to cancer is still barely explored. Our study took advantage of the Electronic Health Records to retrospectively compare the severity of cancer in centenarians (*n* = 649) and non-centenarians (*n* = 62,753) in the Basque Country (province of Gipuzkoa), Spain, through analyzing all the recorded diagnoses throughout their lifetime. Descriptive statistics were applied to discern differences between the two population groups in terms of prevalence of tumor types, number of diagnoses, and treatments. Survival analysis was performed through Kaplan–Meier estimator. We found that centenarians had fewer cancer diagnoses (17.1%, *n* = 111) than non-centenarians (40.5%, *n* = 25,405), and notably avoided the most aggressive cancer types and did not develop metastasis. Furthermore, they barely had records of treatments or drugs and had extended survival both since the first and last diagnosis of cancer. These results suggest resilience of the centenarians against malignant cancers explaining, in part, their extended longevity.

**Supplementary Information:**

The online version contains supplementary material available at 10.1007/s11357-024-01425-4.

## Introduction

Cancer is the second leading cause of death globally, accounting for over 10 million deaths, or 1 in 5 deaths, in 2020. According to the World Health Organization (WHO), lung, prostate, colorectal, and stomach are some of the most common types of cancer in men, whereas breast, colorectal, lung, and cervical are the most common cancers in women [1]. Centenarians have been proposed as a model of healthy aging, and while the reason behind this is presently unclear, they have exhibited potential to avoid or delay major age-related diseases, compressing morbidity until the end of their lives in some cases [2–4]. In regard to cancer, it has been reported that centenarians can delay the onset of cancer by almost 20 years when compared to the rest of the population [5, 6], and few studies have tried to unravel the underlying mechanisms. However, there have been discrepancies regarding the most common tumor types in centenarians across different regions. Among the variety of results, in a few studies, they showed that gastric and lung cancers were the most prevalent in centenarians [7], while other authors found that tumors with higher survival rates such as breast or prostate cancer were the most common ones [5]. These differences could be associated with the different ethnicities, as for instance it has been reported a higher prevalence of gastric cancer in Japanese studies [7].

The Basque Country is one of the most aged regions of Europe, which has been linked to significant multimorbidity and its prevalence increases with age [8]. Regarding cancer, it is the primary cause of death with a mortality rate of 27.4% [9]. Epidemiologic records of Basque Country showed that cancer incidence and cancer-related deaths increased during recent years, being among the higher rates in Europe, but the age-standardized mortality rates decreased [10, 11]. Recently, we characterized the Basque centenarian population and found that they were more resistant to COVID-19, had fewer diseases, and displayed better biological profiles, with lower levels of glucose and triglycerides, among others [12, 13]. In this study, we focused on analyzing the incidence and impact of cancer on Basque centenarians and unraveling the aspects that distinguish them from the rest of the population in the context of this disease.

## Materials and methods

### Study population and design

An observational study retrospectively analyzed real-world data concerning cancer among centenarians. It was based on the data from people age 48 years or older who died between 2014 and 2022 recorded in the database of the Basque Health Service, which contains information from 2004 onward, when the transition to electronic records was performed. Records previous to that date were also stored. All the information registered in the database was anonymized and included demographic and clinical data. Patients who had at least one diagnosis of cancer throughout their lives were studied.

Data quality was evaluated to exclude patients whose data did not meet the quality criteria. Individuals whose date of birth and/or death did not have a valid format (dd/mm/yyyy) or had missing values were excluded, as were patients whose date of birth was after the date of death, or those who had more than one date of death recorded.

After data curation, 63,402 individuals remained. For each individual, information about sex, age, diagnoses, use of resources, and pharmaceutical prescriptions was included. Age was calculated as the difference between the date of death and the date of birth. Non-centenarians were defined as individuals who were younger than 100 years at the time of death (62,723 individuals, 98.93%), and those who were 100 years or older (679 individuals, 1.07%) were centenarians.

The database included information about the diagnoses performed in all the instances of primary care, emergency, outpatient, and in-hospital care and was recorded using diagnosis codes of the International Classification of Diseases, Ninth Revision (ICD-9), and Tenth Revision (ICD-10). Diagnoses of cancer were identified and classified through regular expressions based on ICD-9 codes. Benign neoplasms and records of cancer types with a prevalence of < 0.1% in the population were not considered for the analysis. Diagnoses posterior to patients’ date of death were also discarded. The list of the considered cancer diagnoses is presented in Supplementary Table 1.

The drugs were classified according to the Anatomical Therapeutic Chemical (ATC) code. Antineoplasic prescription records were considered those belonging to group L (antineoplasic agents and immunomodulators) from ATC. The procedures were classified according to the ICD-9 codes using regular expressions. Chemotherapy and radiotherapy admissions and treatment records were considered, even though their inclusion in the Basque Health Service database started in the last years of the study period. The included procedures were also presented in Supplementary Table 1.

### Statistical analyses

Descriptive statistics were applied to analyze the incidence and the numbers and proportions of records of cancer in centenarians and non-centenarians. Survival of both groups since the first and last diagnosis of cancer was assessed using the Kaplan–Meier estimator. Individuals with a single recorded cancer diagnosis were excluded for assessing survival since the last diagnosis. In the survival analysis, individuals deceased after COVID-19 cases started in the Basque Country were also filtered to avoid biased results.

The construction and transformation of the database were performed using Python 3.9. All the statistical analyses were performed using R in RStudio software, v.4.2.2 (URL: https://www.r-project.org/). S*urvival *[14] and *survminer* packages were used for the survival analysis.

### Ethics

This study was approved by the Basque Clinical Research Ethics Committee (CEIm-E code PI2020206) and adhered to the tenets of the Declaration of Helsinki of the World Medical Association on Human Experimentation. The patients and the public were not involved in the design, or conduct, or reporting, or dissemination plans of our research.

## Results

From a cohort of 63,402 individuals deceased between 2014 and 2022 in the population of Gipuzkoa, we studied 25,516 patients who had at least one diagnosis of cancer throughout their lives. Among them, there were 111 centenarians, who represented 17.1% of the total centenarian population, less than half of the proportion of non-centenarian cases (25,405, 40.48% of the total number of non-centenarians) (Table 1). Across the whole population, the distribution of patients who suffered from cancer grew steadily along with age until reaching its highest incidence at 85–90 lifespan, then suddenly decreased (Supplementary Fig. 1). In centenarians, 34 of them developed cancer after reaching 100 years old (30% of the 111 cited above). Of them, 22 (20%) were diagnosed with cancer for the first time after reaching 100 years. Demographic information of the study cohort was included in Supplementary Table 2.

**Table 1:** Incidence of different cancer types in centenarians and non-centenarians. Number of individuals and percentages (%)

Cancer type	Centenarians (*n* = 649)	Non-centenarians (*n* = 62,753)
Any	111 (17.1%)	25,405 (40.48%)
Lip, oral cavity, and pharynx	2 (0.31%)	995 (1.59%)
Stomach	3 (0.46%)	1234 (1.97%)
Colon	7 (1.08%)	2964 (4.72%)
Rectum	3 (0.46%)	1224 (1.95%)
Liver	1 (0.15%)	1141 (1.82%)
Pancreatic	0 (0%)	1257 (2%)
Peritoneum and other digestive organs	3 (0.46%)	1351 (2.15%)
Respiratory and intrathoracic	1 (0.15%)	4412 (7.03%)
Bone	1 (0.15%)	162 (0.26%)
Connective tissue	3 (0.46%)	208 (0.33%)
Skin	50 (7.7%)	3225 (5.14%)
Breast	12 (1.85%)	1920 (3.06%)
Prostate	8 (1.23%)	3006 (4.79%)
Ovary	0 (0%)	391 (0.62%)
Kidney	0 (0%)	1295 (2.06%)
Bladder	5 (0.77%)	2121 (3.38%)
Other genitourinary organs	8 (1.23%)	894 (1.42%)
Brain	1 (0.15%)	662 (1.05%)
Neuroendocrine	0 (0%)	558 (0.89%)
Lymphatic and hematopoietic	3 (0.46%)	1715 (2.73%)
Leukemia	2 (0.31%)	1005 (1.6%)
In situ	13 (2%)	941 (1.5%)
Other locations and not specified	10 (1.54%)	11,034 (17.58%)

When analyzing the incidence of different tumor types, we found that skin cancer was the most prevalent tumor in centenarians (7.7%), while more aggressive cancers such as stomach, liver, brain, lung, and leukemia had an incidence lower than 1% in this population group. It is also remarkable that centenarians completely escaped some malignant neoplasms such as pancreatic cancer. On the other hand, non-centenarians had mostly cancers from other locations and not specified locations, category which included tumors from endocrine glands and located in secondary sites (17.58%). Furthermore, the incidence of tumors that were absent or really scarce in centenarians, such as pancreatic or respiratory cancer, was far higher in non-centenarians (Table 1).

In addition, we analyzed the impact and severity of cancer in both population groups through the number of recorded diagnoses and treatments. Most centenarians had a single diagnosis of cancer (64.86%), a single type of cancer (81.08%), and, remarkably, no record of metastasis. In contrast, most non-centenarians had at least three records of diagnoses of cancer (52.01%), indicating higher monitoring, and there was more variability regarding the numbers of different types of cancer, along with the presence of metastasis records (1.92%). In line with these results, when analyzing the records of prescribed antineoplasic drugs and of chemotherapy and radiotherapy admissions, the proportion of centenarians who had these treatments was far lower than that of non-centenarians (Table 2).

**Table 2:** Comparison of cancer diagnoses and treatments between centenarians and non-centenarians. The information included total number of cancer diagnoses, number of different types of cancer diagnoses, individuals with at least one record of metastasis, antineoplasic drugs, chemotherapy, or radiotherapy. Number of individuals and percentages (%) are presented

	Cancer + centenarians (*n* = 111)	Cancer + non-centenarians (*n* = 25,405)
Diagnoses		
*Number of total diagnoses of cancer*		
* 1*	72 (64.86%)	7099 (27.94%)
* 2*	28 (25.23%)	5093 (20.05%)
* 3*	8 (7.21%)	3948 (15.54%)
> *3*	3 (2.7%)	9265 (36.47%)
*Number of diagnoses of different cancer types*		
* 1*	90 (81.08%)	12,014 (47.29%)
* 2*	18 (16.22%)	9449 (37.19%)
* 3*	2 (1.8%)	3116 (12.27%)
> *3*	1 (0.9%)	826 (3.25%)
* Metastasis*	0 (0%)	489 (1.92%)
Treatments		
* Antineoplasic prescription*	13 (11.71%)	4821 (16.85%)
* Chemotherapy/radiotherapy*	1 (0.9%)	756 (2.98%)

**Fig. 1:**
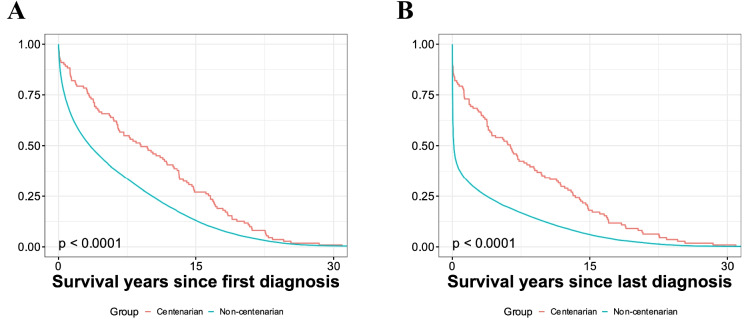
Analysis of survival in cancer patients. **A** Survival of centenarians vs. non-centenarians since the first diagnosis of cancer until death of individuals. **B** Survival of centenarians vs. non-centenarians since the last cancer diagnosis until death of individuals

Next, to deeply assess the centenarians’ response to cancer, we performed different survival analyses. Centenarians showed extended survival from the first diagnosis of cancer until death when compared to non-centenarians *(p* < 0.001) (Fig. 1A). We also studied survival since the last diagnosis of cancer and found that centenarians lived significantly more than non-centenarians, with 50% of them surviving more than 5 years after the last diagnosis, while more than 50% of non-centenarians died in the first year *(p* < 0.001) (Fig. 1B). Furthermore, these differences remained after excluding those individuals who had a single cancer record, which was the case for most centenarians (Supplementary Fig. 2A). Very similar results were obtained after performing the same analysis before the COVID-19 period, to avoid any potential bias (Supplementary Figs. 2B-D).

## Discussion

Studies focused on cancer in centenarians are still scarce [5–7, 15]. The use of Electronic Health Records for analyzing cancer incidence, types, and survival allowed us to characterize centenarians in the context of this disease. We have found that Basque centenarians had a significantly smaller incidence of cancer in comparison with non-centenarians, which agrees with the reports of cancer incidence reaching its peak at 80 years, and drastically decreasing after 100 years [16, 17]. Furthermore, we saw that centenarians were able to escape the most aggressive tumor types, such as liver, lung, or pancreatic, while skin cancer was the most common cancer in this population group, followed by breast, prostate, and other genitourinary system cancers. While some authors supported these findings [5, 18], they also reported that lung and colorectal malignant tumors, which are known as some of the most lethal cancers [19], were among the most common ones in centenarians [18]. However, and in line with our results, other authors reported that in women, who represented the majority of centenarians [12, 20], the incidence and prevalence of melanoma did not present this drastic decrease after 100 years, contrary to the rest types of cancers [21]. Similarly, and along with breast cancer, it did not present such a sudden decrease in mortality; this could hint at extended survival of patients suffering from these tumors when compared to the rest types of cancer [21]. Coherently, in our survival analysis, we saw that since their first diagnosis of cancer, centenarians lived more than non-centenarians, which once again could be associated with them having less aggressive tumors [5]. These findings are also in line with those that stated that mortality rates of neoplasms decrease from 40% in old individuals to 4% in centenarians, whose causes of death were mostly associated with circulatory system diseases [22].

Interestingly, as the number of records related to cancer in the database was far lower for centenarians, this potential resistance could not be linked to a more exhaustive monitoring from healthcare systems. In fact, our analysis of diagnoses and treatment records suggests that centenarians did not spend much time with cancer, since most of them had a single diagnosis and very few of them had treatments recorded. Even though chemotherapy treatments may not always be feasible in the oldest old [23], the patterns of the survival curves showed that centenarians did better than the rest of the population, living several years after both first and last diagnoses. In addition, the null metastatic rate exhibited by centenarians in our database suggests a possible resistance against cancer dissemination. While it is true that non-melanoma skin cancer has a very low rate of metastasis [24], breast and prostate cancers have been reported as some of the malignant tumors with the highest metastatic rates [25]; therefore, centenarians do not seem to only elude metastasis-prone tumors, but they are also resilient to metastasis when they suffered from tumors prone to it. Studies that explore metastasis in centenarians are scarce; however, it has been reported that the metastatic rate for centenarians is 38%, lower than the 51% of individuals aged 90–94 [26]. These percentages, even though far higher than the ones found in our database due to the scarce of records, still agree with the differences reported.

All in all, these results reinforce those that postulate centenarians as a resilient age group in concern to cancer [26], explaining in part their extreme longevity.

This study has some limitations. The reduced number of cancer diagnoses in centenarians impaired the use of statistics and the stratification in the survival analysis by cancer types. It is possible that some centenarians died with an undetected cancer, even though this could also be the case of non-centenarians. Similarly, we considered metastasis only in those cases in which it was specified by the ICD code corresponding to neoplasm morphology. It is possible that some cancer records did not include this type of information, and therefore, the reflected incidence was probably lower for both groups. However, the neoplasm category “Other locations and not specified” also includes metastatic tumors, among others, and we found a clear increase in the incidence of this category in non-centenarians compared to centenarians. The database did not include the specific causes of death; therefore, we could not establish a direct association between cancer and mortality. Furthermore, the records of chemotherapy and radiotherapy treatments were not included since the beginning of the study period and therefore the reflected incidence was probably lower. There could also be cases in which the frail status of individuals discouraged the treatment with chemotherapy, which could have happened specially in the case of centenarians who developed cancer in the last years of their lives. In line with this, since we did not have information about which surgical procedures were due to cancer, we did not include this data when addressing cancer treatments. Overall, the retrospective analysis of Electronic Health Records, which were implemented in 2004, could have produced a bias regarding older records that were not correctly registered or that changed their format throughout years.

## Supplementary Information

Below is the link to the electronic supplementary material.

Supplementary file1 (DOCX 2097 KB)

## Data Availability

The original contributions presented in the study are included in the article/Supplementary material; further inquiries can be directed to the corresponding author.
