# Supplementary material for: Centenarians of the Basque Country are resilient to cancer
Source: GeroScience. 2024 Nov 9;47(2):2309–15. doi: 10.1007/s11357-024-01425-4 (PMC11979019; doi:10.1007/s11357-024-01425-4)
Supplement: Supplementary file 1 — Supplementary file1 (DOCX 2097 KB) [file 11357_2024_1425_MOESM1_ESM.docx]

**Supplementary File S1**

**Supplementary Table 1. List of ICD-9 codes included in the analysis**

**Supplementary Table 2. Demographic characteristics of the study cohort**

**Supplementary Figure S1.** **Distribution of cancer patients**

**Supplementary Figure S2.** **Analysis of survival of cancer patients**

**Supplementary Table 1. List of ICD-9 codes included in the analysis.**

| **ICD-9 codes** |  |
| --- | --- |
| **Neoplasm diagnoses** | |
| 140-149 | Lip, oral cavity, and pharynx |
| 150, 152, 156, 158, 159 | Peritoneum and other digestive organs |
| 151 | Stomach |
| 153 | Colon |
| 154 | Rectum |
| 155 | Liver |
| 157 | Pancreatic |
| 160-165 | Respiratory and intrathoracic |
| 170 | Bone |
| 171 | Connective tissue |
| 172, 173, 176 | Skin |
| 174-175 | Breast |
| 185 | Prostate |
| 183 | Ovary |
| 189 | Kidney |
| 188 | Bladder |
| 179-182, 184, 186-187 | Other genitourinary organs |
| 190, 192-199 | Other locations and not specified |
| 191 | Brain |
| 200-203 | Lymphatic and hematopoietic |
| 204-208 | Leukemia |
| 209 | Neuroendocrine |
| 210-229 | Benign |
| 230-234 | In situ |
| 235-238 | Uncertain evolution |
| 239 | Not specified nature |
| **Contact with health services** | |
| V580, V581 | Admission for radiotherapy or chemotherapy |
| **Procedures** | |
| 922-924, 9925, 9928 | Radiotherapy and chemotherapy |

|  | **CENTENARIANS**  **(n = 649)** | | **NON-CENTENARIANS**  **(n = 62,753)** | |
| --- | --- | --- | --- | --- |
|  | **CANCER+**  **(n = 111)** | **CANCER-**  **(n = 538)** | **CANCER+**  **(n = 25,405)** | **CANCER-**  **(n = 37,348)** |
| **Demographic data** |  | | | |
| Age | 102.03 ± 1.91 | 102.07 ± 1.9 | 78.81 ± 11.44 | 84.17 ± 10.63 |
| Sex | 89 women  (80.18%)  22 men  (19.82%) | 468 women  (86.99%)  70 men  (13.01%) | 10,182 women  (40.08%)  15,223 men  (59.92%) | 20,738 women  (55.53%)  16,610 men  (44.47%) |
| Residence | 31 yes  (27.93%)  80 no  (72.07%) | 156 yes  (29%)  382 no  (71%) | 2,245 yes  (8.84%)  23,160 no  (91.16%) | 6,715 yes  (17.98%)  30,633 no  (82.02%) |

**Supplementary Table 2. Demographic characteristics of the study cohort.**

**
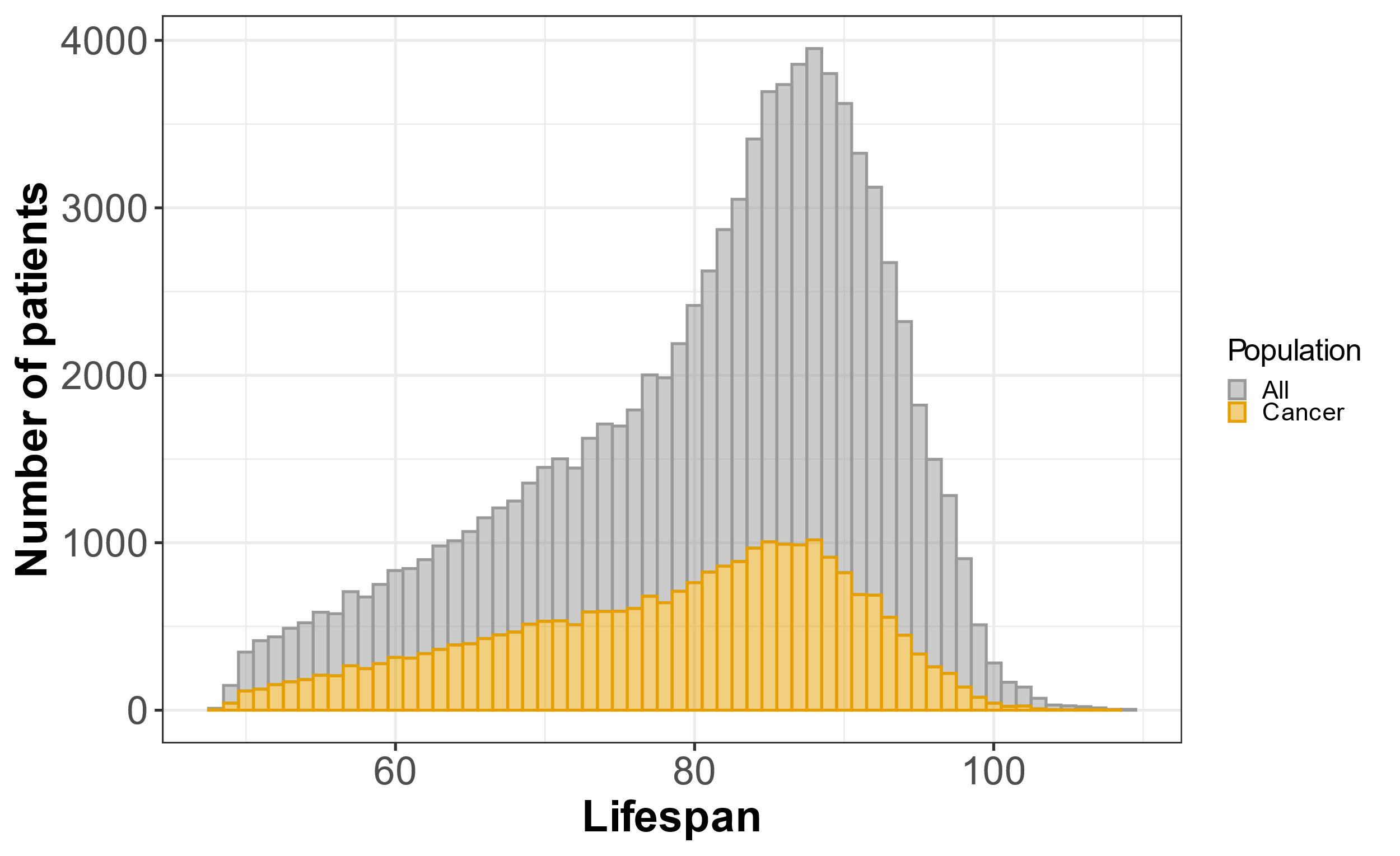
Distribution of cancer patients**

**Supplementary Figure Caption S1.** **Distribution of cancer patients.**

**Analysis of survival of cancer patients**


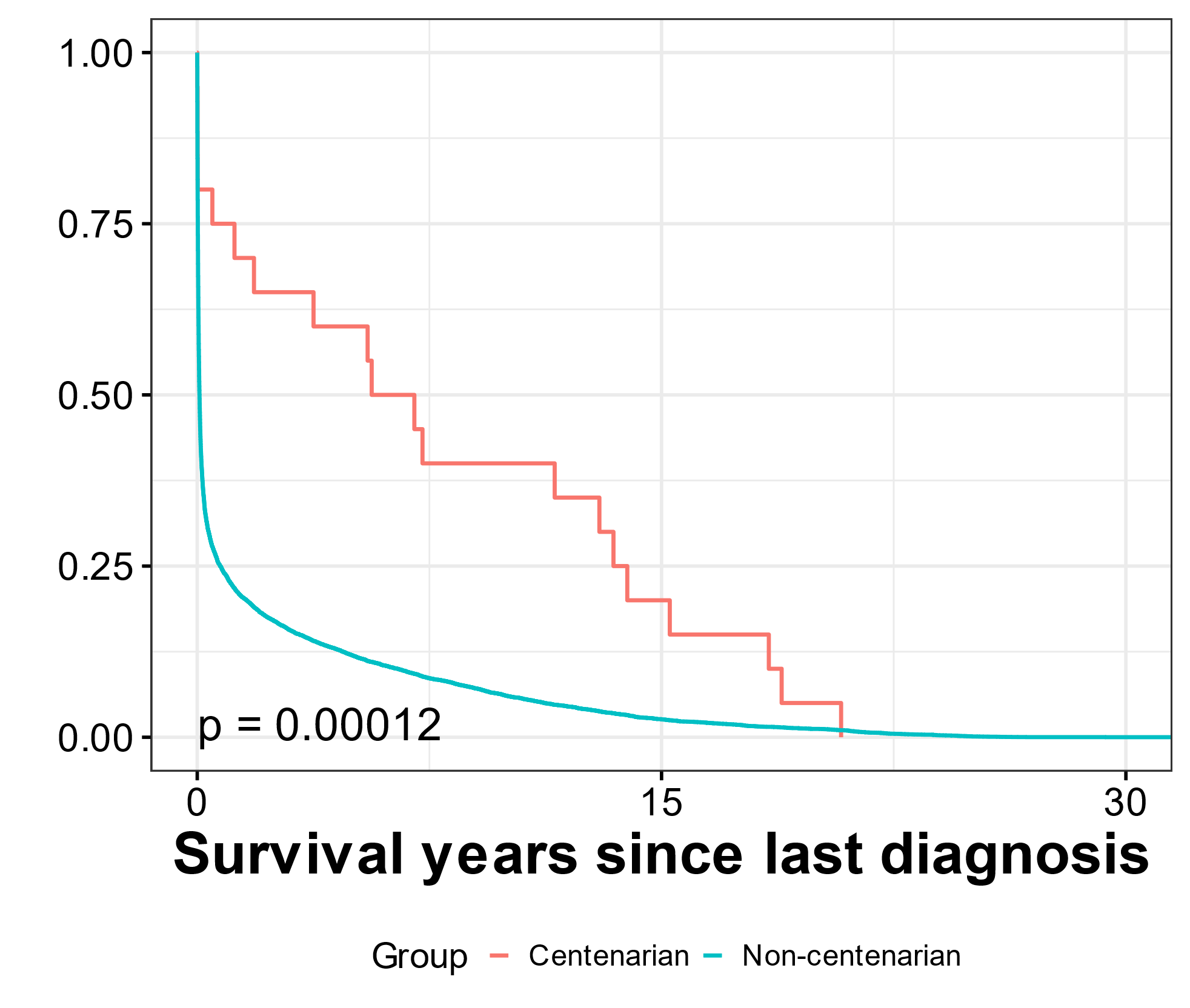

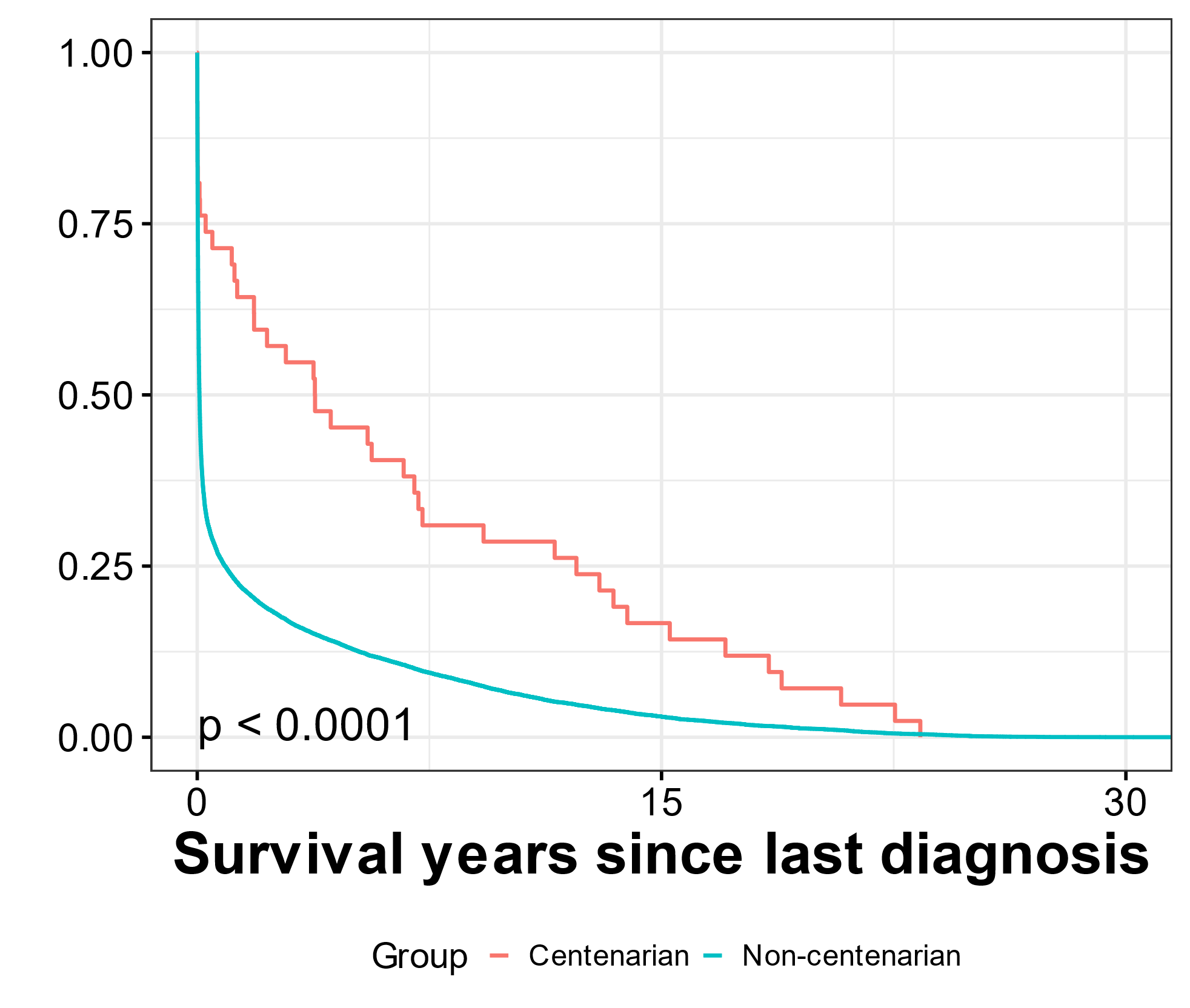

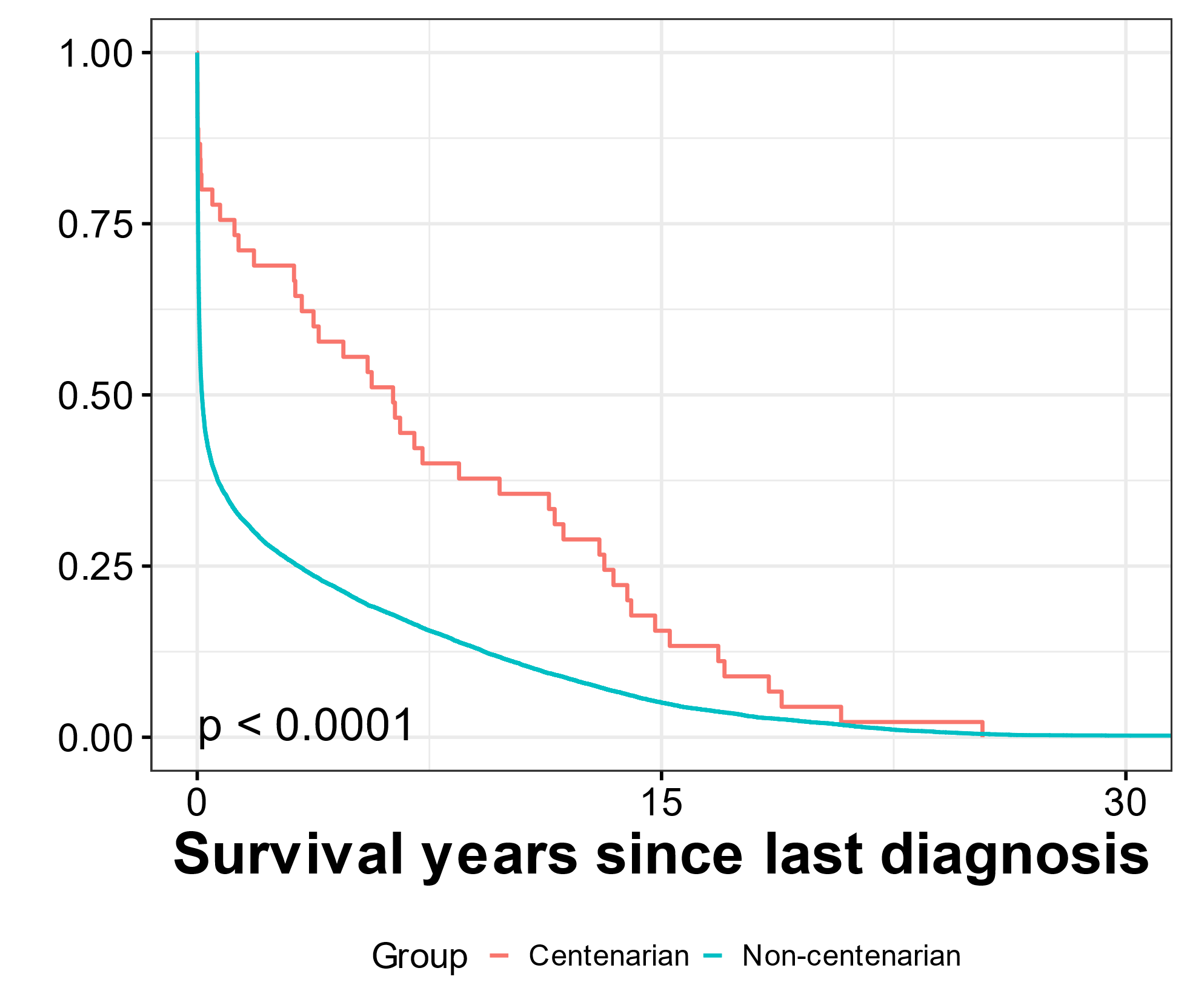

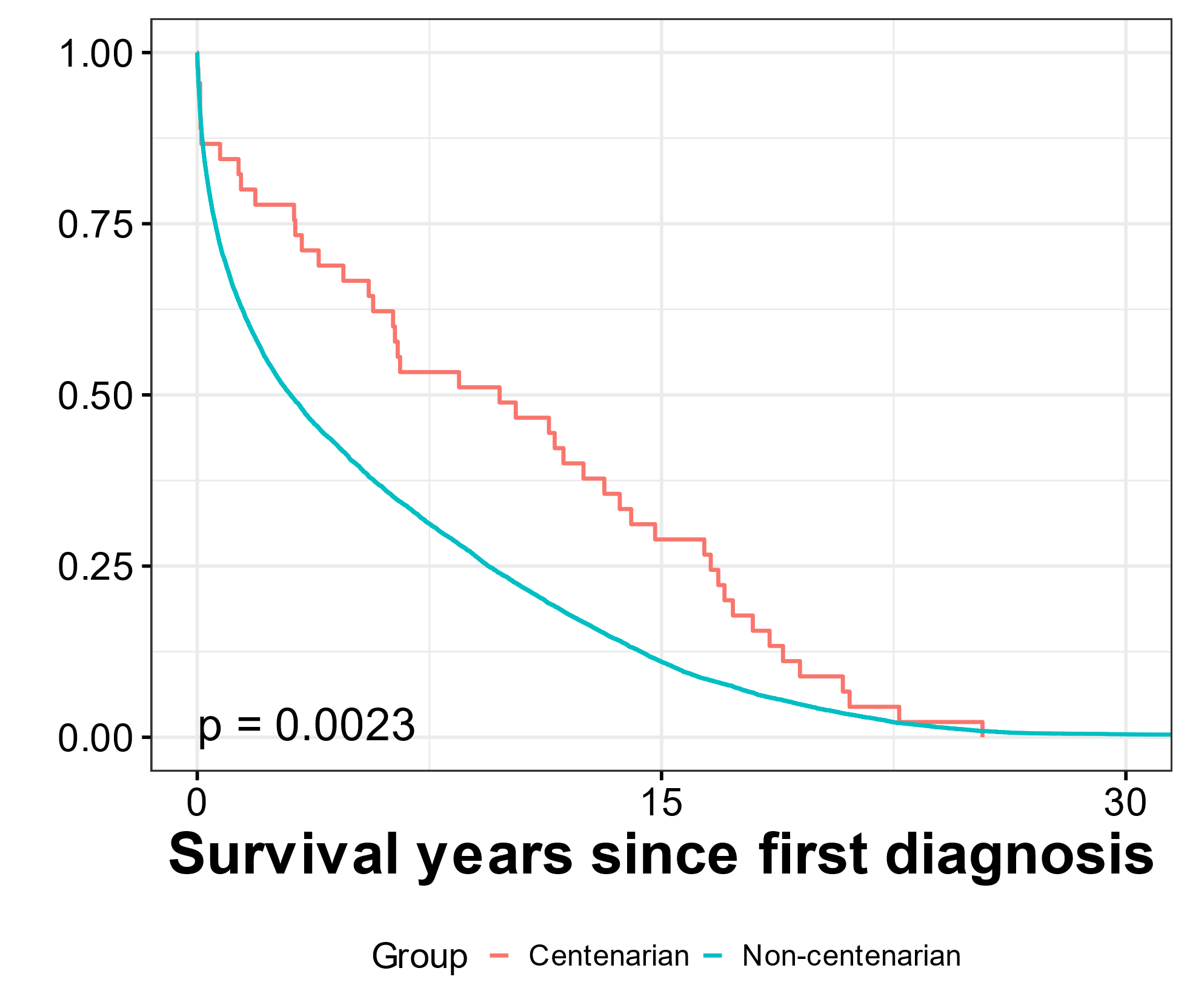


**A**

**B**

**C**

**D**

**Supplementary Figure Caption S2.** **Analysis of survival of cancer patients. A)** Survival of centenarians vs non-centenarians since the last cancer diagnosis until death of individuals. Only individuals with more than one cancer diagnosis were considered. **B)** Survival of centenarians vs non-centenarians since the first cancer diagnosis until death of individuals. Only individuals deceased before COVID-19 pandemic were considered. **C)** Survival of centenarians vs non-centenarians since the last cancer diagnosis until death of individuals. Only individuals deceased before COVID-19 pandemic were considered. **D)** Survival of centenarians vs non-centenarians since the last cancer diagnosis until death of individuals. Only individuals with more than one cancer diagnosis deceased before COVID-19 pandemic were considered.
